# Quality of life and functional outcomes after low anterior rectal resection with total mesorectal excision: a propensity score-matched comparison of robotic-assisted versus open surgery

**DOI:** 10.1007/s00423-026-04314-w

**Published:** 2026-10-05

**Authors:** Frederick Bröcker, Susanne Merkel, Anne Jacobsen, Klaus Weber, Axel Denz, Robert Grützmann, Georg F. Weber, Maximilian Brunner, Christian Krautz

**Affiliations:** 1 Department of Surgery, University Hospital Erlangen, Friedrich-Alexander-Universität Erlangen-Nürnberg (FAU), Krankenhausstraße 12, 91054 Erlangen, Germany; 2 Comprehensive Cancer Center Erlangen–European Metropolitan Area of Nürnberg (CCC ER- EMN), Erlangen, Germany

**Keywords:** Rectal cancer, Total mesorectal excision, Robotic surgery, Quality of life, LARS, Patient-reported outcomes

## Abstract

**Background:**

Robotic-assisted surgery (RAS) for rectal cancer is increasingly adopted. However, a recent systematic review highlighted a significant gap in the literature regarding quality of life (QOL) after different total mesorectal excision (TME) techniques, with open TME being particularly underrepresented. This study aimed to compare QOL and anorectal function between robotic-assisted and open low anterior rectal resection with TME.

**Methods:**

Patients who underwent low anterior rectal resection with TME and primary anastomosis between 2006 and 2021 at a single tertiary centre were identified. Propensity score matching was performed based on sex, age, BMI, UICC stage and tumour height. After excluding patients without QOL follow-up or with a persistent diverting stoma, 40 patients per group were available. QOL was assessed using the EORTC QLQ-C30, QLQ-CR29, and LARS score.

**Results:**

In the matched cohort, baseline characteristics were well balanced except for tumour height (*p* = .002), with more lower-third tumours in the open group. No significant differences were observed in any EORTC QLQ-C30 domain, including global health status (66.5 vs. 71.7, *p* = .197). QLQ-CR29 showed comparable outcomes for most domains; however, sexual interest was significantly lower in the RAS group for women (88.1 vs. 55.6, *p* = .008). LARS scores were similar between groups (24.4 vs. 25.6, *p* = .765). Perioperative outcomes including morbidity (10.0% vs. 22.5%, *p* = .130) and anastomotic leakage (2.5% vs. 5.0%, *p* = .526) did not differ significantly.

**Conclusions:**

Robotic-assisted and open low anterior rectal resection with TME resulted in comparable QOL and anorectal function across all assessed domains. Notably, approximately one third of patients in both groups reported major LARS, underscoring the persistent functional burden irrespective of surgical approach. By providing direct comparative data on open TME – an approach underrepresented in the literature – these findings contribute to closing an important evidence gap.

## Introduction

Rectal cancer surgery has evolved substantially over the past decades, with total mesorectal excision (TME) established as the oncological standard for curative resection. While open surgery has long been the conventional approach, minimally invasive techniques including laparoscopic and robotic-assisted surgery (RAS) have increasingly been adopted, with the aim of reducing surgical trauma while maintaining oncological outcomes [1, 2].

Robotic-assisted surgery offers potential advantages including three-dimensional high-definition visualisation, enhanced instrument articulation, tremor filtration, and improved surgeon ergonomics [3]. These technical features may facilitate precise dissection within the confined pelvic space, potentially leading to improved nerve preservation and better functional outcomes. However, disadvantages include higher costs, absence of haptic feedback, and a relevant learning curve [1, 4].

While short-term perioperative outcomes of robotic rectal surgery have been extensively studied, evidence on patient-reported outcomes including quality of life (QOL) and anorectal function remains limited. The few available studies comparing functional outcomes after different surgical approaches for rectal cancer have yielded heterogeneous results, partly attributable to differences in study design, patient selection, and follow-up intervals [5–7]. Notably, Veltcamp Helbach et al. reported comparable QOL outcomes between laparoscopic and transanal TME, although their analysis was limited by significant differences in follow-up duration and anastomosis type [5].

The landmark ROLARR trial comparing robotic and laparoscopic rectal surgery found no significant difference in conversion rates but was not designed to assess functional outcomes [2]. More recently, the REAL trial, a multicentre randomised study of 1,240 patients, demonstrated that robotic surgery significantly reduced locoregional recurrence (1.6% vs. 4.0%, *p* = .03) and improved disease-free survival compared to laparoscopic surgery, with better functional outcomes particularly during the first postoperative year [8]. However, these trials compared robotic with laparoscopic approaches. A systematic review and meta-analysis by Geitenbeek et al. evaluated functional outcomes across all four TME techniques (open, laparoscopic, robotic, and transanal) in 2,495 patients and found no significant differences in urinary, sexual, or fecal functioning [9]. Critically, open TME was represented by only 88 patients (3.5%), highlighting a significant evidence gap [9]. A network meta-analysis of 29 randomised trials (6,237 patients) by Phan et al. similarly concluded that all TME techniques result in comparable perioperative morbidity and long-term survival, although minimally invasive approaches may improve postoperative recovery [10]. Neither review identified robust evidence for QOL superiority of any technique over open surgery. This study aimed to address this evidence gap by comparing QOL and functional outcomes between robotic-assisted and open low anterior rectal resection with TME using validated patient-reported outcome measures in a propensity score-matched cohort from a single high-volume tertiary centre.

## Materials and methods

### Study design and patient selection

This study was reported in accordance with the STROBE statement for observational studies. This retrospective single-centre study included patients who underwent elective low anterior rectal resection with TME and primary anastomosis for histologically confirmed rectal carcinoma at the Department of Surgery, University Hospital Erlangen, between 2006 and 2021. Inclusion criteria were: confirmed rectal carcinoma treated by low anterior rectal resection with TME and primary anastomosis, patient alive at time of follow-up, and documented BMI. Exclusion criteria were familial adenomatous polyposis (FAP), chronic inflammatory bowel disease (CIBD), and conversion or extension of the surgical procedure.

**Fig. 1:**
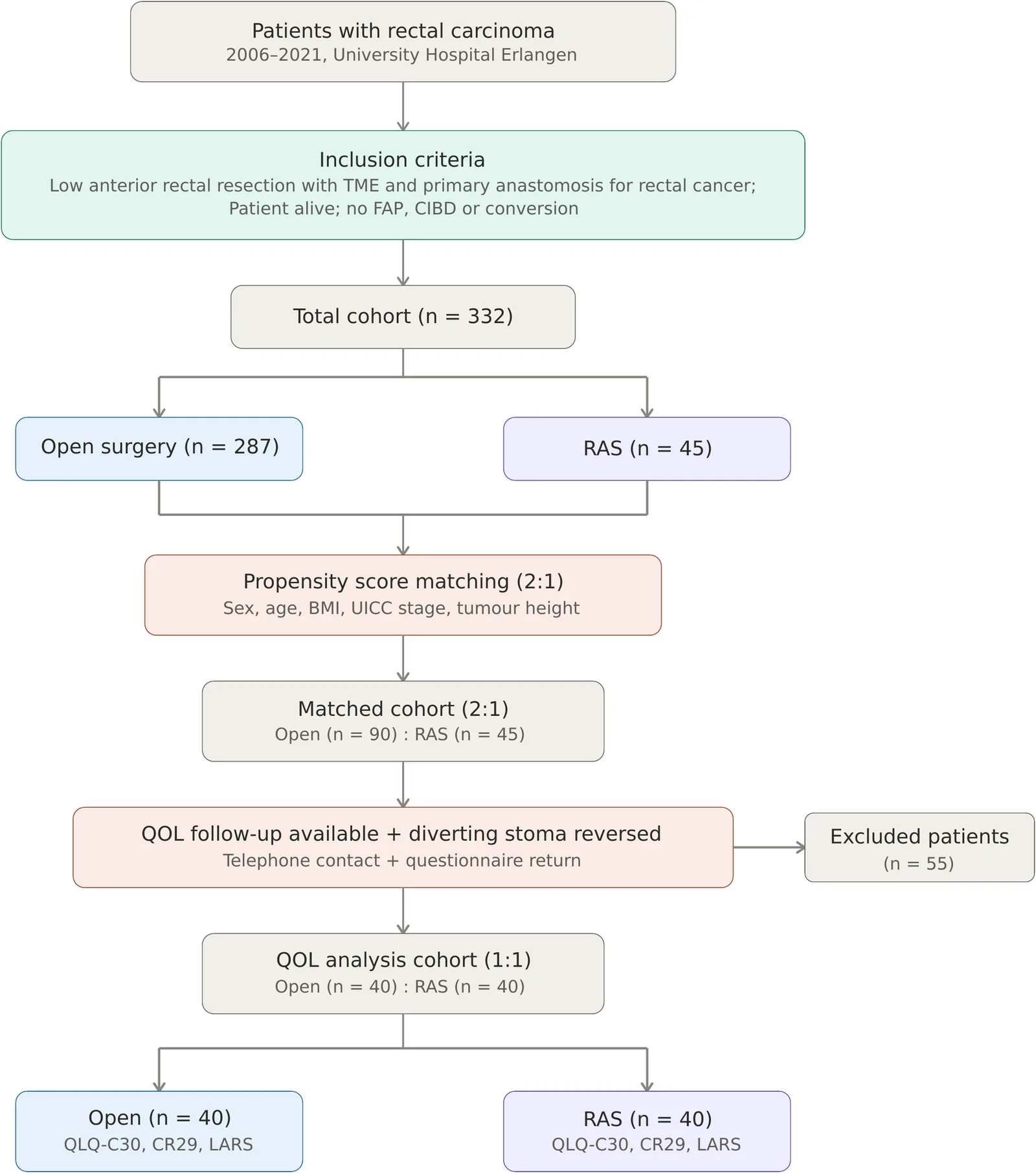
Patient inclusion and matching process. This flowchart shows the process of selecting patients for propensity score matching and forming the final quality-of-life analysis cohort for comparing robot-assisted surgery (RAS) with open surgery in cases of rectal cancer. Of the 332 eligible patients, 45 patients undergoing RAS were matched with 90 patients undergoing open surgery at a ratio of 2:1 according to sex, age, BMI, UICC stage and tumour height. Following the exclusion of patients without quality-of-life follow-up or repositioned diverting stoma, 40 patients remained in each group for the final analysis

A total of 287 patients who underwent open surgery and 45 patients who underwent robotic-assisted surgery were identified. Robotic-assisted procedures were performed using the da Vinci Surgical System (Intuitive Surgical, Sunnyvale, CA, USA), which was introduced at our institution in 2012. Consequently, the open cohort includes patients operated from 2006 onwards, whereas all robotic-assisted procedures were performed between 2012 and 2021. Notably, laparoscopic TME was not routinely performed at our institution during the study period, resulting in a direct institutional transition from open to robotic-assisted surgery. From 2012 onwards, the choice between the open and robotic-assisted approach was based on surgeon preference and robot availability, and not on patient-related factors such as insurance status. Propensity score matching was performed based on the following variables: sex, age at surgery, body mass index (BMI), UICC tumour stage, and tumour height from the anal verge. From the matched cohort, only patients who were alive, had their diverting stoma reversed, and returned completed questionnaires were eligible for the QOL analysis, resulting in 40 patients per group (*n* = 80) (Fig. 1).

The study was conducted in accordance with the Declaration of Helsinki and approved by the local ethics committee of the Friedrich-Alexander-Universität Erlangen-Nürnberg [26-219-Br]. All patients provided written informed consent for the use of their clinical data and participation in the QOL follow-up.

### Surgical procedures

All patients underwent low anterior rectal resection with TME. Open surgery was performed through laparotomy. Robotic-assisted procedures were performed using the da Vinci Surgical System (Intuitive Surgical, Sunnyvale, CA, USA). Both approaches included TME to ensure complete removal of the mesorectal envelope with its lymphatic drainage. All anastomoses were performed as end-to-end anastomoses.

### Neoadjuvant therapy

Neoadjuvant treatment was administered according to German S3 guidelines for rectal cancer. Depending on tumour stage and localisation, patients received no neoadjuvant therapy, chemoradiotherapy (CRT), total neoadjuvant therapy (TNT, consisting of induction chemotherapy followed by CRT), short-course radiotherapy (5 × 5 Gy), or induction chemotherapy alone.

### Functional assessment

All patients were contacted by telephone and subsequently received three validated questionnaires by mail: the EORTC QLQ-C30 (version 3.0), the EORTC QLQ-CR29, and the LARS score questionnaire. QOL assessment was performed as a single cross-sectional evaluation per patient, reflecting the current QOL status at the time of contact. Because of the extended inclusion period of the open cohort, the interval between surgery and QOL assessment differed substantially between groups (see Results, Table 1).

**Table 1:** Patient demographics and surgical details

	Total cohort			Matched cohort		
	Open (*n* = 287)	RAS (*n* = 45)	*p*-value	Open (*n* = 40)	RAS (*n* = 40)	*p*-value
Age at surgery (years), median (IQR)	60 (52–68)	60 (54–69)	0.491	60 (55–69)	60 (54–68)	0.810
Sex, n (%)			0.263			1.000
Male	166 (57.8%)	22 (48.9%)		25 (62.5%)	25 (62.5%)	
Female	121 (42.2%)	23 (51.1%)		15 (37.5%)	15 (37.5%)	
BMI (kg/m²), median (IQR)	26.0 (23.7–28.7)	25.1 (23.0–27.2)	0.169	25.4 (22.3–28.1)	25.3 (23.2–26.9)	0.773
ASA, n (%)			0.176			0.265
1	59 (20.6%)	4 (8.9%)		9 (22.5%)	4 (10.0%)	
2	198 (69.0%)	36 (80.0%)		25 (62.5%)	31 (77.5%)	
3	30 (10.5%)	5 (11.1%)		6 (15.0%)	5 (12.5%)	
Neoadjuvant therapy, n (%)			0.065			0.402
None	124 (43.2%)	22 (48.9%)		18 (45.0%)	18 (45.0%)	
CRT	152 (53.0%)	18 (40.0%)		21 (52.5%)	17 (42.5%)	
CRT + Ind. CT (TNT)	7 (2.4%)	3 (6.7%)		1 (2.5%)	3 (7.5%)	
Short-course RT (5 × 5 Gy)	2 (0.7%)	0 (0.0%)		0 (0.0%)	0 (0.0%)	
Induction CT only	2 (0.7%)	2 (4.4%)		0 (0.0%)	2 (5.0%)	
Time from surgery to survey, months, median (IQR)				153.8 (98.4–167.3)	45.6 (29.7–55.0)	< 0.001
Age at survey (years), median (IQR)				71 (66–79)	63 (55–68)	< 0.001
Tumor height^a^			0.012			0.002
Lower third	54 (18.8%)	1 (2.2%)		11 (27.5%)	1 (2.5%)	
Middle third	170 (59.2%)	29 (64.4%)		25 (62.5%)	27 (67.5%)	
Upper third	63 (22.0%)	15 (33.3%)		4 (10.0%)	12 (30.0%)	
Temporary diverting stoma, n (%)	228 (79.4%)	37 (82.2%)	0.416	35 (87.5%)	32 (80.0%)	0.648

*IQR* interquartile range, *BMI* body mass index, *ASA *american society of anesthesiologists, ^a^Tumor height: cm from anal verge, Lower = 0–<6 cm, Middle = 6–<12 cm, Upper = 12–16 cm [11]

#### EORTC QLQ-C30

This instrument comprises a global health status/QOL scale, five functional scales (physical, role, emotional, cognitive, social), three multi-item symptom scales (fatigue, nausea/vomiting, pain), and six single-item symptom scales. Scores are transformed to a 0–100 scale according to the EORTC scoring manual [12]. Higher scores on functional scales and global health status indicate better functioning; higher scores on symptom scales indicate greater symptom burden.

#### EORTC QLQ-CR29

This colorectal cancer-specific module comprises four functional scales (anxiety, weight, body image, sexual interest) and symptom scales including urinary, gastrointestinal, and sexual function domains. Scoring follows the same principles as the QLQ-C30 [13]

#### LARS score

This five-item questionnaire assesses flatus incontinence, liquid stool incontinence, bowel frequency, clustering of stools, and urgency. Total scores classify patients as no LARS (0–20), minor LARS (21–29), or major LARS (30–42) [14].

### Statistical analysis

Propensity score matching was performed using SPSS (IBM SPSS Statistics, version 28). Continuous variables are reported as median with interquartile range (IQR); categorical variables are reported as absolute numbers and percentages. Group comparisons were performed using the Mann–Whitney U test for continuous variables and the chi-squared test or Fisher’s exact test for categorical variables. The Shapiro–Wilk test confirmed non-normal distribution of QOL data, supporting the use of non-parametric testing. Differences in mean QoL scores larger than 10 points were considered clinically meaningful [15]. No a priori sample size calculation was performed; the cohort size was determined by the available patients meeting the inclusion criteria.

## Results

### Baseline characteristics

In the total cohort (*n* = 332), the open group comprised 287 patients and the RAS group 45 patients. After propensity score matching and selection of patients with available QOL data, 40 patients per group were included in the matched cohort. Baseline characteristics of both the total and matched cohort are presented in Table 1.

In the matched cohort, age (median 60 years in both groups, *p* = .810) and BMI (median 25.4 vs. 25.3 kg/m², *p* = .773) were well balanced. Sex distribution was identical (62.5% male in both groups). ASA classification did not differ significantly (*p* = .265). The median interval from surgery to QOL assessment differed substantially between groups (153.8 months [IQR 98.4–167.3] vs. 45.6 months [IQR 29.7–55.0], *p* < .001), reflecting the earlier inclusion period of the open cohort. In addition, tumour height distribution remained significantly imbalanced (*p* = .002), with more lower-third tumours in the open group (27.5% vs. 2.5%) despite tumour height being a matching variable. This is explained by the fact that the propensity score matching initially yielded a larger matched cohort, which was subsequently reduced based on QOL follow-up availability, potentially disrupting the initially achieved balance.

### Perioperative and oncological outcomes

Perioperative and pathological outcomes are presented in Table 2. TME quality was comparable between groups, with complete TME in 92.5% (open) and 87.5% (RAS) of matched cases. All resections achieved R0 status. The (y)pT category differed significantly (*p* = .039), with more pT3 tumours in the RAS group (42.5% vs. 20.0%); this is explained by the fact that UICC stage (not pT) was the matching variable, allowing for different pT/pN compositions within balanced overall stages. Nodal status was comparable (*p* = .762). Overall morbidity was numerically higher in the RAS group (22.5% vs. 10.0%, *p* = .130) without reaching statistical significance. Anastomotic leakage rates were low in both groups (2.5% vs. 5.0%, *p* = .526).

**Table 2:** Outcome parameters

	Total cohort			Matched cohort		
	Open (*n* = 287)	RAS (*n* = 45)	*p*-value	Open (*n* = 40)	RAS (*n* = 40)	*p*-value
Completeness TME, n (%)			0.737			0.712
Complete	241 (84.0%)	40 (88.9%)		37 (92.5%)	35 (87.5%)	
Nearly complete	36 (12.5%)	5 (11.1%)		3 (7.5%)	5 (12.5%)	
Incomplete	3 (1.0%)	0 (0.0%)		0 (0.0%)	0 (0.0%)	
T category, n (%)			0.039			0.039
(y)pT0/1/2	203 (70.7%)	23 (51.1%)		32 (80.0%)	21 (52.5%)	
(y)pT3	82 (28.6%)	20 (44.4%)		8 (20.0%)	17 (42.5%)	
(y)pT4	2 (0.7%)	1 (2.2%)		0 (0.0%)	1 (2.5%)	
N category, n (%)			0.956			0.762
(y)pN0	235 (81.9%)	37 (82.2%)		33 (82.5%)	34 (85.0%)	
(y)pN+	52 (18.1%)	8 (17.8%)		7 (17.5%)	6 (15.0%)	
Resection margin, n (%)			–			–
R0	287 (100%)	45 (100%)		40 (100%)	40 (100%)	
Morbidity, n (%)	41 (14.3%)	10 (22.2%)	0.170	4 (10.0%)	9 (22.5%)	0.130
Clavien–Dindo, n (%)			0.640			0.574
I	9 (3.1%)	3 (6.7%)		2 (5.0%)	3 (7.5%)	
II	17 (5.9%)	4 (8.9%)		1 (2.5%)	3 (7.5%)	
IIIa	6 (2.1%)	2 (4.4%)		1 (2.5%)	2 (5.0%)	
IIIb	7 (2.4%)	1 (2.2%)		0 (0.0%)	1 (2.5%)	
IVa	2 (0.7%)	0 (0.0%)		0 (0.0%)	0 (0.0%)	
Anastomotic leakage, n (%)	6 (2.1%)	2 (4.4%)	0.311	1 (2.5%)	2 (5.0%)	0.526
Re-surgery, n (%)	9 (3.1%)	1 (2.2%)	0.739	0 (0.0%)	1 (2.5%)	0.314

*TME* total mesorectal excision, *TME* completeness data were unavailable for 7 patients in the open total cohort

### Quality of life: EORTC QLQ-C30

QOL outcomes assessed by the QLQ-C30 are presented in Table 3. No significant differences were observed between the open and RAS groups in any of the functional or symptom scales. Global health status/QOL was numerically higher in the RAS group (71.7 vs. 66.5) without reaching significance (*p* = .197). Functional scale scores were comparable across all domains, with social functioning showing the largest numerical difference (68.3 vs. 75.0, *p* = .242). Symptom scales were similarly balanced, with diarrhoea showing the largest numerical difference (29.9 vs. 22.5, *p* = .197).

**Table 3:** EORTC QLQ-C30 summary (matched cohort)

QoL Parameter	Open Mean (SD)	RAS Mean (SD)	*p* value
***Symptom scales*** ^a^			
Fatigue	23.89 (27.74)	26.39 (28.54)	0.709
Nausea and vomiting	1.67 (7.36)	3.33 (13.71)	0.423
Pain	18.33 (22.58)	12.92 (22.80)	0.199
Dyspnoea	14.53 (23.93)	10.83 (21.86)	0.412
Insomnia	25.00 (29.96)	26.67 (33.93)	0.950
Appetite loss	9.17 (21.33)	8.33 (24.75)	0.431
Constipation	15.00 (26.10)	10.83 (20.52)	0.717
Diarrhoea	29.91 (28.40)	22.50 (28.63)	0.197
Financial difficulties	12.82 (31.17)	15.83 (28.23)	0.463
***Functional scales*** ^b^			
Physical functioning	84.50 (16.29)	86.62 (14.87)	0.471
Role functioning	74.58 (26.95)	75.83 (23.55)	0.944
Emotional functioning	77.75 (22.23)	76.88 (25.63)	0.895
Cognitive functioning	85.62 (19.70)	87.08 (19.05)	0.763
Social functioning	68.33 (28.19)	75.00 (28.49)	0.242
***Global health status/QoL*** ^**b**^	66.46 (21.14)	71.67 (20.66)	0.197

^a^ Higher score indicates worse health-related quality of life, ^b^ Higher score indicates better health-related quality of life

### Quality of life: EORTC QLQ-CR29

Colorectal cancer-specific QOL outcomes are presented in Table 4. Most symptom and functional domains were comparable between groups. Two notable findings emerged in the functional scales: sexual interest was significantly lower in the RAS group for women (88.1 vs. 55.6, *p* = .008) and showed a borderline difference for men (68.2 vs. 49.3, *p* = .050). Among symptom scales, buttock pain showed a non-significant numerical difference disfavouring the RAS group (11.7 vs. 20.8, *p* = .073). Faecal incontinence, urinary frequency, and other gastrointestinal symptoms were comparable.

**Table 4:** EORTC QLQ-CR29 summary (matched cohort)

QoL Parameter	Open Mean (SD)	RAS Mean (SD)	*p* value
***Symptom scales*** ^a^			
Urinary frequency	40.83 (22.31)	32.50 (26.41)	0.129
Urinary incontinence	15.38 (25.18)	9.17 (16.86)	0.277
Dysuria	1.67 (7.36)	1.67 (10.54)	0.579
Abdominal pain	8.33 (16.45)	11.97 (22.28)	0.622
Buttock pain	11.67 (24.52)	20.83 (28.93)	0.073
Bloating	20.83 (27.93)	25.00 (24.75)	0.315
Blood and mucus in stool	2.08 (5.58)	4.58 (9.98)	0.311
Dry mouth	15.83 (21.33)	15.00 (27.16)	0.471
Hair loss	8.55 (22.58)	6.67 (21.62)	0.508
Taste	7.50 (20.66)	5.00 (19.32)	0.317
Flatulence	44.95 (27.79)	41.88 (33.09)	0.558
Faecal incontinence	34.31 (34.31)	23.93 (27.52)	0.215
Sore skin	18.09 (28.40)	24.79 (31.27)	0.335
Stool frequency	38.57 (29.08)	39.32 (28.99)	0.908
Embarrassment	29.52 (34.08)	28.20 (37.88)	0.621
Impotence	69.45 (37.16)	53.97 (38.70)	0.213
Dyspareunia	13.33 (32.20)	16.67 (33.33)	0.872
***Functional scales*** ^b^			
Anxiety	70.00 (27.01)	71.67 (27.79)	0.772
Weight	82.50 (25.02)	79.17 (23.49)	0.372
Body image	83.20 (19.47)	77.78 (28.24)	0.678
Sexual interest (men)	68.18 (26.18)	49.28 (31.58)	0.050
Sexual interest (women)	88.10 (16.57)	55.55 (29.59)	0.008

^a^ Higher score indicates worse health-related quality of life, ^b^ Higher score indicates better health-related quality of life

### Anorectal function: LARS score

LARS outcomes are presented in Table 5. Mean LARS scores were nearly identical (24.4 vs. 25.6, *p* = .765). The distribution across LARS categories was similar, with major LARS present in 37.8% (open) and 37.5% (RAS) of patients. No significant differences were observed in any individual LARS item, including incontinence for flatus (*p* = .423), incontinence for liquid stools (*p* = .395), bowel frequency (*p* = .619), clustering of stools (*p* = .396), and urgency (*p* = .836).

**Table 5:** LARS questionnaire summary (matched cohort)

LARS	Open (*n* = 37)	RAS (*n* = 40)	*p* value
Incontinence for flatus			0.423
Never	14	15	
< Once a week	10	8	
≥ Once a week	13	17	
Incontinence for liquid stools			0.395
Never	14	19	
< Once a week	14	15	
≥ Once a week	9	6	
Bowel frequency			0.619
> 7 times a day (24 h)	4	3	
4–7 times a day (24 h)	9	16	
1–3 times a day (24 h)	17	18	
< Once a day (24 h)	7	3	
Clustering of stools			0.396
Never	5	3	
< Once a week	11	11	
≥ Once a week	21	26	
Urgency			0.836
Never	14	14	
< Once a week	14	16	
≥ Once a week	9	10	
LARS categories			
No	13	12	
Minor	10	13	
Major	14	15	
LARS (mean, 95% CI)	24.35	25.55	0.765

*LARS* low anterior resection syndrome, *No* score 0–20, *Minor* score 21–29, *Major * score 30–42

### Learning curve subanalysis

To explore a potential impact of the institutional learning curve on QOL outcomes, the 46 robotic-assisted cases with available QOL data were divided chronologically by operative date into an early group (first 23 cases; November 2012 to March 2019) and a late group (last 23 cases; March 2019 to November 2021). Numerical improvements were observed in the late group across all three QOL summary scores: mean Global QoL (66.7 vs. 73.9), mean functional scales (77.0 vs. 84.3), and mean symptom scales (19.2 vs. 10.6; lower values indicating fewer symptoms). None of these differences reached statistical significance (all *p* > .20 by Mann–Whitney U test), and Spearman correlation of case number with QOL outcomes was likewise non-significant (Global QoL: ρ = +0.148, *p* = .32; functional: ρ = +0.190, *p* = .20; symptom: ρ = − 0.113, *p* = .45). Furthermore, the numerical between-group difference in Global QoL (approximately 7 points) did not exceed the pre-specified minimally important difference of 10 points [15]. These findings do not support a clinically or statistically meaningful effect of the institutional learning curve on the QOL outcomes reported in this cohort.

## Discussion

This study compared QOL and functional outcomes between robotic-assisted and open low anterior rectal resection with TME in a propensity score-matched cohort from a single high-volume centre. The principal finding is that both surgical approaches yielded comparable patient-reported outcomes across all major QOL domains and anorectal function measures.

Our findings are consistent with the conclusions of two recent comprehensive reviews. Geitenbeek et al. conducted a systematic review and meta-analysis of 19 studies encompassing 2,495 patients and found no significant differences in urinary, sexual, or fecal functioning or QOL across open, laparoscopic, robotic, and transanal TME techniques [9]. Notably, open TME was represented by only 88 patients, underscoring the paucity of QOL data for this approach. Similarly, a network meta-analysis of 29 RCTs by Phan et al. (6,237 patients) concluded that all techniques result in comparable perioperative morbidity and long-term survival [10]. Neither review identified evidence for QOL superiority of any minimally invasive technique over open surgery. Our propensity score-matched comparison adds to this limited evidence base by providing a direct comparison of RAS and open TME with three standardised QOL instruments, confirming the absence of clinically meaningful QOL differences. Regarding specific QOL domains, our results align with existing data. Veltcamp Helbach et al. compared laparoscopic and transanal TME and similarly found no major QOL differences, although their analysis identified significant differences in role functioning, fatigue, and financial difficulties that were attributed to unequal follow-up durations (59.5 vs. 20.0 months) [5]. The LARS scores in our cohort (mean 24.4 and 25.6 for open and RAS, respectively) are comparable to those reported in the literature, with major LARS rates of approximately 38% in both groups. Long-term data from the Dutch TME trial have shown that up to 46% of patients experience major LARS as far as 14 years after surgery [8], indicating that bowel dysfunction remains a significant burden regardless of surgical technique.

The Global QOL scores in our cohort (66.5 and 71.7, open vs. RAS, respectively) are comparable to the 12-month values reported in the EDIUM study (66 points), a large multicentre German patient-reported outcomes study of 3,142 surgically treated colorectal cancer patients in certified cancer centres [16]. This suggests that the functional outcomes in our cohort are within the expected range for this patient population, despite the considerably longer follow-up interval in the open group.

The REAL trial recently provided high-level evidence that robotic surgery offers functional advantages over laparoscopic surgery, with significantly better urinary, sexual, and defecation function during the first postoperative year [8]. However, these benefits were observed against laparoscopic – not open – surgery. Compared with open surgery, robotic-assisted TME would be expected to offer even more pronounced advantages, given the substantial ergonomic and visualization limitations of open access to the deep pelvis, particularly in male and obese patients. The absence of a QOL advantage for RAS over open surgery in our cohort therefore warrants explanation. Several factors likely contributed. The considerably longer follow-up interval in the open group (median 153.8 vs. 45.6 months) may have allowed functional recovery and psychological adaptation to narrow initial differences (see Limitations). Both approaches were performed at a high-volume tertiary centre by experienced surgeons, and our exploratory chronological subanalysis did not demonstrate a statistically significant learning-curve effect on QOL in the robotic cohort (see Results), suggesting that technique-related differences were mitigated by surgeon expertise. Finally, the open group contained a higher proportion of lower-third tumours – a group known to have worse functional outcomes – which would, if anything, have disfavoured the open cohort, reinforcing the validity of the observed comparability.

The significantly lower sexual interest among women in the RAS group (*p* = .008) is the only statistically significant QOL difference in our analysis and warrants careful interpretation. The subgroup sizes were small (*n* = 14 and *n* = 12), limiting the reliability of this finding, and as an isolated result among numerous comparisons a type I error due to multiple testing cannot be excluded. Moreover, sexual function is known to recover over time after rectal surgery, and the substantially longer postoperative interval in the open group may partly explain their superior scores. Future studies should incorporate standardised follow-up time points and sufficient sample sizes for subgroup analyses of sexual function.

A relevant methodological consideration pertains to the persistent imbalance in tumour height despite its inclusion as a matching variable. The RAS group contained only one patient with a lower-third tumour (2.5%) compared to 11 patients (27.5%) in the open group. This structural imbalance may be a result of the fact that the propensity score matching initially yielded a larger matched cohort, which was subsequently reduced based on QOL follow-up availability, potentially disrupting the initially achieved balance for this variable. Re-matching after this reduction was not pursued, as it would have further decreased the sample size below the threshold required for meaningful statistical comparison. However, the functional relevance of this imbalance is mitigated by the fact that only patients who underwent low anterior resection with TME were included in this study. While PME is generally an option for upper rectal tumours [17, 18], patients with upper-third tumours in our cohort underwent TME, most likely due to locally advanced tumour stages (cT3/4) necessitating complete mesorectal excision to ensure adequate circumferential and distal resection margins [19, 20]. Importantly, it is the anastomotic height – rather than tumour height per se – that primarily determines functional outcomes. Patients with less than 4 cm of remnant rectum have a 46% risk of major LARS compared to 10% in those with 4 cm or more [21, 22], and a recent meta-analysis confirmed that TME carries a significantly higher risk of major LARS compared to PME (OR 0.34, *p* < .001) [23, 24]. Since all patients in our study underwent TME with a low pelvic anastomosis – as evidenced by the high rates of protective diverting stomas in both groups (87.5% and 80.0%) – the functional risk profile was comparable between groups, irrespective of primary tumour location. Nevertheless, the RAS group’s more favourable tumour height distribution would, if anything, be expected to result in better functional outcomes. The fact that QOL outcomes were comparable rather than superior in the RAS group suggests that robotic-assisted surgery did not confer a measurable functional advantage in this cohort.

The significantly higher proportion of pT3 tumours in the RAS group (42.5% vs. 20.0%, *p* = .039) requires explanation. Matching was performed on overall UICC stage, not on individual T or N categories. Consequently, balanced UICC stages can comprise different pT/pN combinations across groups. This finding does not indicate a relevant imbalance in oncological burden between groups.

Several limitations of this study should be acknowledged. First, the retrospective design and single-centre setting limit generalisability. Second, the sample size of 40 patients per group restricts the ability to detect clinically significant differences. It is important to note that the absence of statistical significance should not be interpreted as evidence of equivalence; a formal non-inferiority design would be required to draw such a conclusion. Third, the follow-up intervals for QOL assessment differed substantially between groups (median 153.8 vs. 45.6 months, *p* < .001), reflecting the introduction of robotic-assisted surgery at our institution in 2012 and the consequently earlier inclusion period of the open cohort. This is a major limitation of our analysis. A meaningful statistical correction for the follow-up interval would have required longitudinal QOL data, which were not available. Quality of life after rectal cancer surgery is known to change over time, with initial impairment followed by gradual recovery and psychological adaptation. Our finding of comparable QOL between groups must therefore be interpreted with this temporal confounder in mind. Fourth, perioperative parameters such as operative time, blood loss, and length of hospital stay were not included, as the primary focus of this study was on patient-reported QOL outcomes. Fifth, survival and recurrence data were deliberately excluded to avoid confounding the QOL analysis with oncological outcomes that may affect patient well-being independently. Finally, this study did not include EQ-5D or IPSS assessments, which would have provided further insight into generic health-related quality of life and urinary function.

Strengths of this study include the use of propensity score matching to reduce selection bias, the application of three validated and complementary QOL instruments, and the inclusion of both a total and matched cohort analysis. The single-centre design ensures standardised surgical techniques and perioperative care pathways across both groups.

## Conclusion

In this exploratory propensity score-matched cohort from a single high-volume centre, robotic-assisted and open low anterior rectal resection with TME were associated with comparable quality of life and functional outcomes. However, the substantial difference in follow-up intervals between groups – with the open cohort assessed at a median of more than 10 years after surgery – represents a major confounder, as long-term psychological adaptation may mitigate initial functional impairment. The observed difference in sexual interest among women should be interpreted with caution given the small subgroup sizes and differing follow-up intervals. These data contribute to the limited body of evidence on functional outcomes following open versus robotic-assisted rectal resection, and should be regarded as hypothesis-generating within the limited evidence base for open TME. As minimally invasive techniques continue to evolve and institutions gain more experience, prospective studies with standardised follow-up are needed to better understand the effect of surgical technique on long-term quality of life after rectal cancer surgery.

## Data Availability

All data supporting the findings of this study are available within the paper and its Supplementary Information.

## Declarations

### Use of artificial intelligence

Artificial intelligence (Claude, Anthropic) was used for language editing and literature search assistance. All scientific content, data analysis, interpretation, and conclusions are the sole responsibility of the authors.
